# Metformin reverses bFGF-induced epithelial-mesenchymal transition in HCC cells

**DOI:** 10.18632/oncotarget.22200

**Published:** 2017-10-31

**Authors:** Wang Chengye, Tian Yu, Shao Ping, Sun Deguang, Wang Keyun, Wang Yan, Zhang Rixin, Liang Rui, Gao Zhenming, Ye Mingliang, Wang Liming

**Affiliations:** ^1^ Division of Hepatobiliary and Pancreatic Surgery, Department of Surgery, The Second Hospital of Dalian Medical University, Dalian, Liaoning 116023, China; ^2^ CAS Key Lab of Separation Sciences for Analytical Chemistry, National Chromatographic Research and Analysis Center, Dalian Institute of Chemical Physics, Chinese Academy of Sciences, Dalian, Liaoning 116023, China

**Keywords:** hepatocellular carcinoma, metformin, basic fibroblast growth factor, epithelial-mesenchymal transition, Twist1

## Abstract

Metformin had exerted important inhibitory effects in multiple cancers. However, the correlation between metformin and hepatocellular carcinoma (HCC) metastasis, and the relevant mechanisms are still unclear. By quantitative proteomics analysis technique, we found metformin could suppress FGF signalling significantly. In FGF signalling basic fibroblast growth factor (bFGF) is a crucial member, it initially binds to its receptors, the complex of bFGF and receptors activate FGF signallings, and promote many cancers progressions. When treating HCC cell lines HepG2 and Huh7 with bFGF, we observed the cells exhibited epithelial mesenchymal transition (EMT) and these cells metastasis potential was enhanced dramaticlly. However, when treating with metformin and bFGF together, EMT and metastasis induced by bFGF could be inhibited in these cells. Furthermore, bFGF could activate AKT/GSK-3β signalling, sequentially decrease the interaction between GSK-3β and Twist1 and decrease ubiquitination of Twist1 leading to Twist1 degradation reducing. While metformin could repress the bFGF-mediated activation in AKT/GSK-3β signalling, inhibition on interaction between GSK-3β and Twist1, enhancement of Twist1 stability. Taken together, our findings suggested that metformin had prominent negative effects on bFGF-induced EMT and metastasis in HCC cells.

## INTRODUCTION

Liver cancer has been the second leading cause of cancer-related mortality for a long time throughout the world, and approximately 50% of the total number of new cases and deaths is from China [1]. Although the incidence and mortality of liver cancer have decreased in China, it is still the leading cause of death and the most common cancer in men younger than 60 years of age [2]. Hepatocellular carcinoma (HCC), which originates from hepatic cells, accounts for 85-90% of total primary liver cancers [3]. Despite many emerging therapies, such as transarterial chemoembolization, radioembolization, radiofrequency ablation and molecular targeted therapy, surgical excision is still the main therapeutic strategy in HCC, but the rate of recurrence after surgical resection of HCC is approximately 50-70%. One of the main reasons for this is the high rate of intrahepatic and/or extrahepatic metastasis [4]. Therefore, developing new techniques or medicines to prevent metastasis will clearly benefit the treatment of HCC.

Epithelial-mesenchymal transition (EMT) was initially described in embryonic development in the early 1980s [5], and it participates in multiple processes such as tumour progression, ageing, inflammation and development [6–9]. Epithelial cells adhere to and communicate with one another via intercellular junctions including tight junctions, gap junctions and desmosomes. During EMT, epithelial cells transdifferentiate into mesenchymal cells accompanied by losing cellular junctions and epithelial cell-specific phenotypes such as E-cadherin and acquiring N-cadherin and Vimentin, which are hallmarks of mesenchymal cells [7, 10, 11]. As EMT endows epithelial cells mesenchymal characteristics leading to migration and invasion of cancer cells, this process has been suggested as being one of the vitally important mechanisms in cancer metastasis [12]. During EMT, transcriptional factors including Snail, Twist and ZEB, which are regulated by multiple signalling pathways [13], such as the PI3K/AKT, ERK/MAPK [14, 15], Wnt/β-catenin [16] pathways, have been demonstrated to have crucial roles [17, 18]. Among these, Twist1, which is a basic helix-loop-helix transcriptional factor, promotes EMT by downregulating E-cadherin. Twist1 can recruit several necessary proteins to constitute a complex that binds to the E-box sequence at the E-cadherin promoter and represses its transcriptional activity, thereby decreasing E-cadherin expression [19, 20]. However, the exact impact of Twist1 in EMT has not been identified.

As a family member of fibroblast growth factors (FGFs), basic fibroblast growth factor (bFGF) and its receptors have been reported to play vital roles in many physiological and pathological processes including visceral organ development, angiogenesis and malignant progression of cancer [21, 22]. bFGF binds to and activates its tyrosine kinase receptors, leading to the activation of downstream signalling pathways and subsequently regulating gene expression or DNA replication to affect the physiological and pathological status of cells [23]. Accumulating evidence has confirmed that bFGF participates in the progression of HCC. Motoaki et al. demonstrated that bFGF could enhance the proliferation and motility of cancer cells in HCC through autocrine mechanisms [24]. During cancer metastasis, bFGF was also shown to induce EMT through the AKT/GSK-3β/Snail signalling pathway, resulting in the promotion of migration and invasion of prostate cancer cells [25]. Although it has been reported that bFGF can induce EMT of the HCC cell line Huh7 [26], the mechanisms have not been elucidated.

Metformin, an oral anti-hyperglycaemic agent, is widely used to treat type 2 diabetes mellitus (T2DM). Metformin improves insulin sensitivity in peripheral tissues of T2DM patients and inhibits hepatic gluconeogenesis, resulting in the diminution of glycaemia. Recently, Morales et al. reported that metformin could dramatically decrease both the incidence and mortality of multiple cancers [27]. In addition, metformin was demonstrated to decrease invasion by preventing EMT in cancers [28, 29], these data suggest that metformin could be a potential adjuvant agent in cancer treatment. However, its detailed mechanisms as an anti-cancer agent remain unclear.

In the present study, we showed that bFGF could induce EMT and that metformin could reverse bFGF-induced EMT in HepG2 and Huh7 cells. Additionally, bFGF promoted EMT by altering the interaction between GSK-3β and Twist1, resulting in the enhanced stability of Twist1 mediated by the AKT/GSK-3β signalling pathway. Furthermore, the inhibition of bFGF-induced EMT by metformin was also mediated through the regulation of the AKT/GSK-3β/Twist1 pathway.

## RESULTS

### bFGF induced EMT in HepG2 and Huh7 cells promoting metastasis

**Figure 1:**
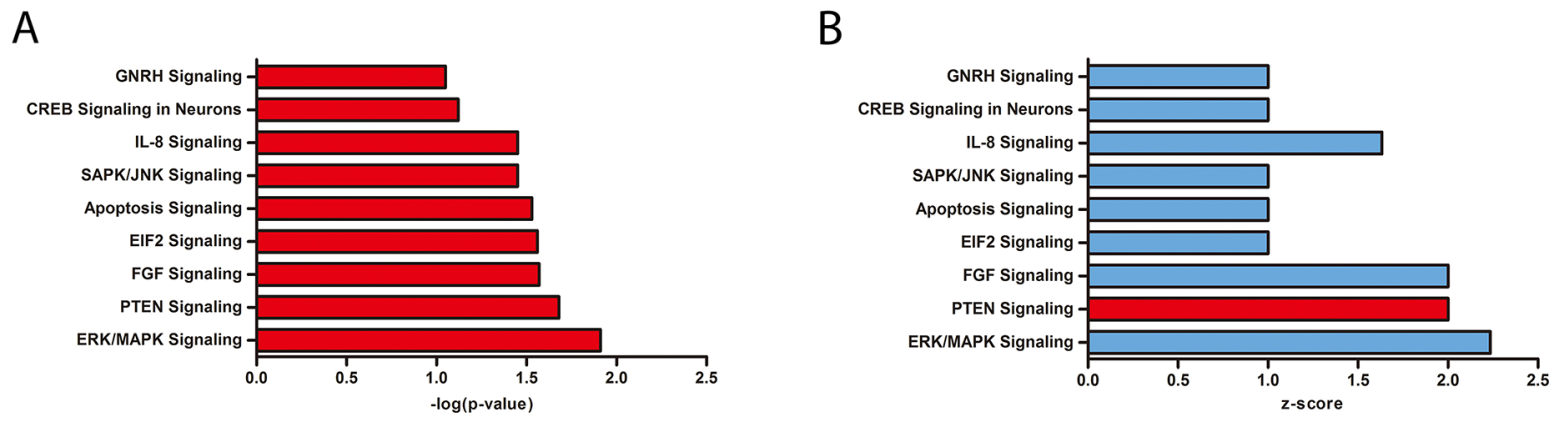
Quantitative proteomics highlighted differences between normal control and metformin-treated HCC cells by Ingenuity Pathway Analysis **(A)** –log(p-value) of Ingenuity Pathway Analysis. **(B)** z-score of Ingenuity Pathway Analysis. Blue<0, red>0.

To elucidate the key molecules and/or signalling pathways in metformin-mediated inhibition of HCC, we determined the differences in proteins expression of HCC cells with or without metformin (10 μmol/ml) after 48 hours of treatment using quantitative proteomics. A total of 2707 proteins were quantified, and among these, 430 proteins were found to have differential expression, including 52 upregulated [deuterium/hydrogen ratio (D/H)>1.5] and 378 downregulated proteins (D/H<0.67, Supplementary Table 1). The Ingenuity Pathway Analysis (IPA) tool was further adopted for grouping the proteins into networks and canonical pathways to determine the pathways influenced by metformin in HCC. We found several pathways that had been altered, and we focused on the FGF signalling pathway, which was dramatically inhibited by metformin treatment (Figure 1).

**Figure 2:**
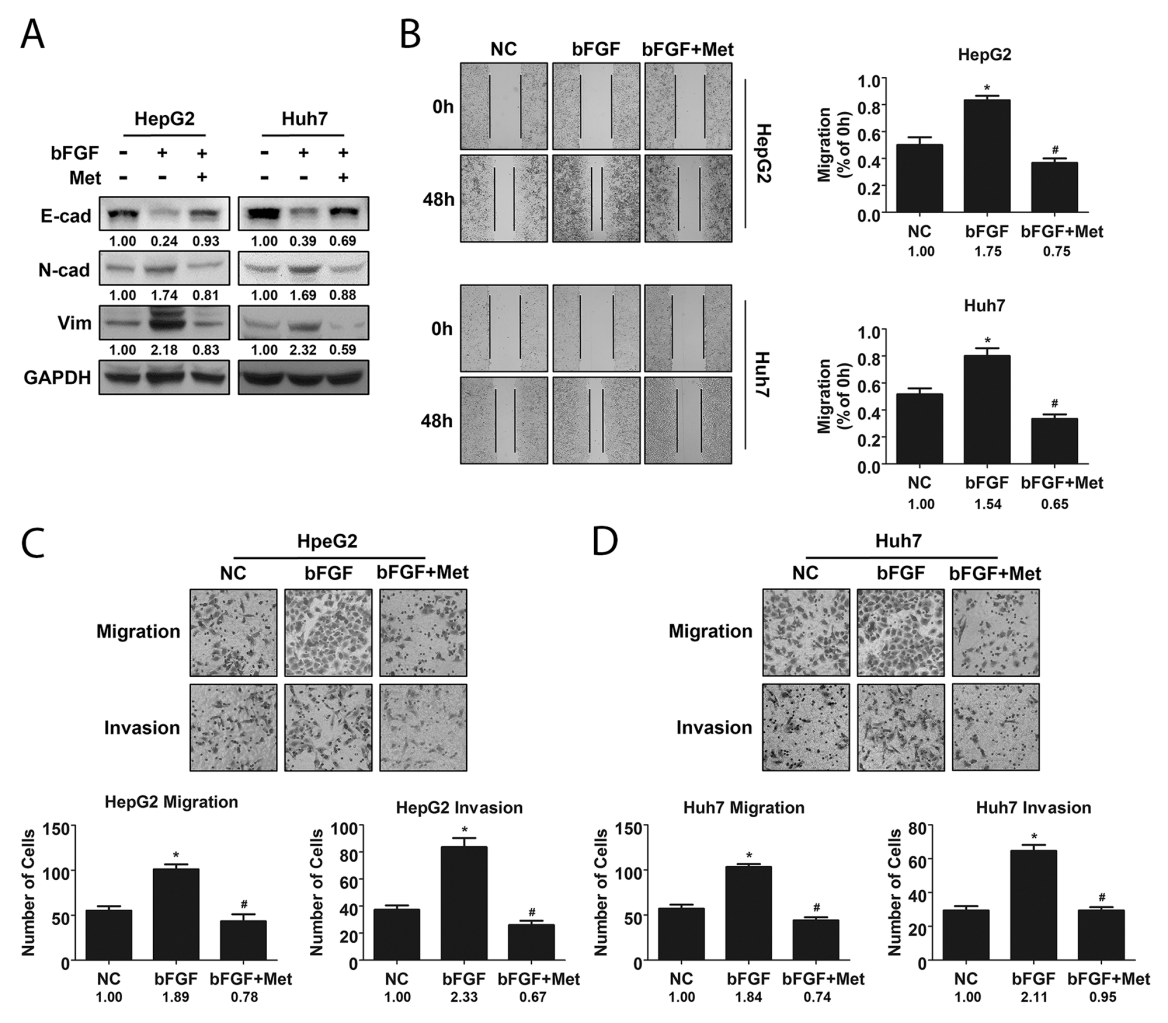
Metformin reversed EMT induced by bFGF in HepG2 and Huh7 cells **(A)** Expression of phenotypic proteins including E-cad, N-cad and Vimentin in cells treated without and with bFGF (20 μg/ml) or bFGF (20 μg/ml) and metformin (10 μmol/ml) together was analysed by western blotting. **(B)** Metastatic potential of cells treated without and with bFGF or bFGF and metformin together was evaluated by wound healing scratch assay. **(C, D)** Migration and invasion of cells treated without and with bFGF or bFGF and metformin together were assessed using transwell assay. ^*^p<0.05 compared with the control, ^#^p<0.05 compared with bFGF treatment.

bFGF is a growth factor that interacts with fibroblast growth factor receptor to activate FGF signalling. In HCC, bFGF was shown to regulate motility of cancer cells to promote metastasis [24]. As EMT plays an important role in metastasis, we wondered whether metformin could suppress the FGF signalling pathway stimulated by bFGF in HCC EMT. To confirm our hypothesis, we selected HepG2 and Huh7 cells to study the effects of bFGF and metformin on EMT in HCC. After treating cells with bFGF (20 ng/ml) for 48 h, we detected changes in phenotypes of cells cultured with bFGF, including the upregulation of mesenchymal markers N-cadherin and Vimentin, and the downregulation of the epithelial marker E-cadherin compared to those in the control. In addition, after treatment with bFGF (20 μg/ml) and metformin (10 μmol/ml) together for 48 h, mesenchymal properties of the cells were reverted to epithelial characteristics (Figure 2A). In accordance with the phenotypic changes in cells, the wound area of cells treated with bFGF was significantly narrower than that of cells without treatment, and the effect of bFGF was reversed after the cells were cultured with bFGF and metformin together as shown by the wound healing assay (Figure 2B). Similar phenomena were observed by migration and invasion assays, where the number of migrated and invaded cells was higher in response to bFGF treatment; however, after being treated by bFGF and metformin together for 48 h, the number of migrated and invaded cells showed no obvious differences with that of cells without treatment, indicating that metformin reversed the bFGF-mediated induction of EMT (Figure 2C-2D). These data indicated that bFGF could induce EMT in HepG2 and Huh7 cells, while metformin exerted negative effects on EMT induced by bFGF in HepG2 and Huh7 cells.

### bFGF induced EMT in HCC cells by upregulating Twist1 expression

**Figure 3:**
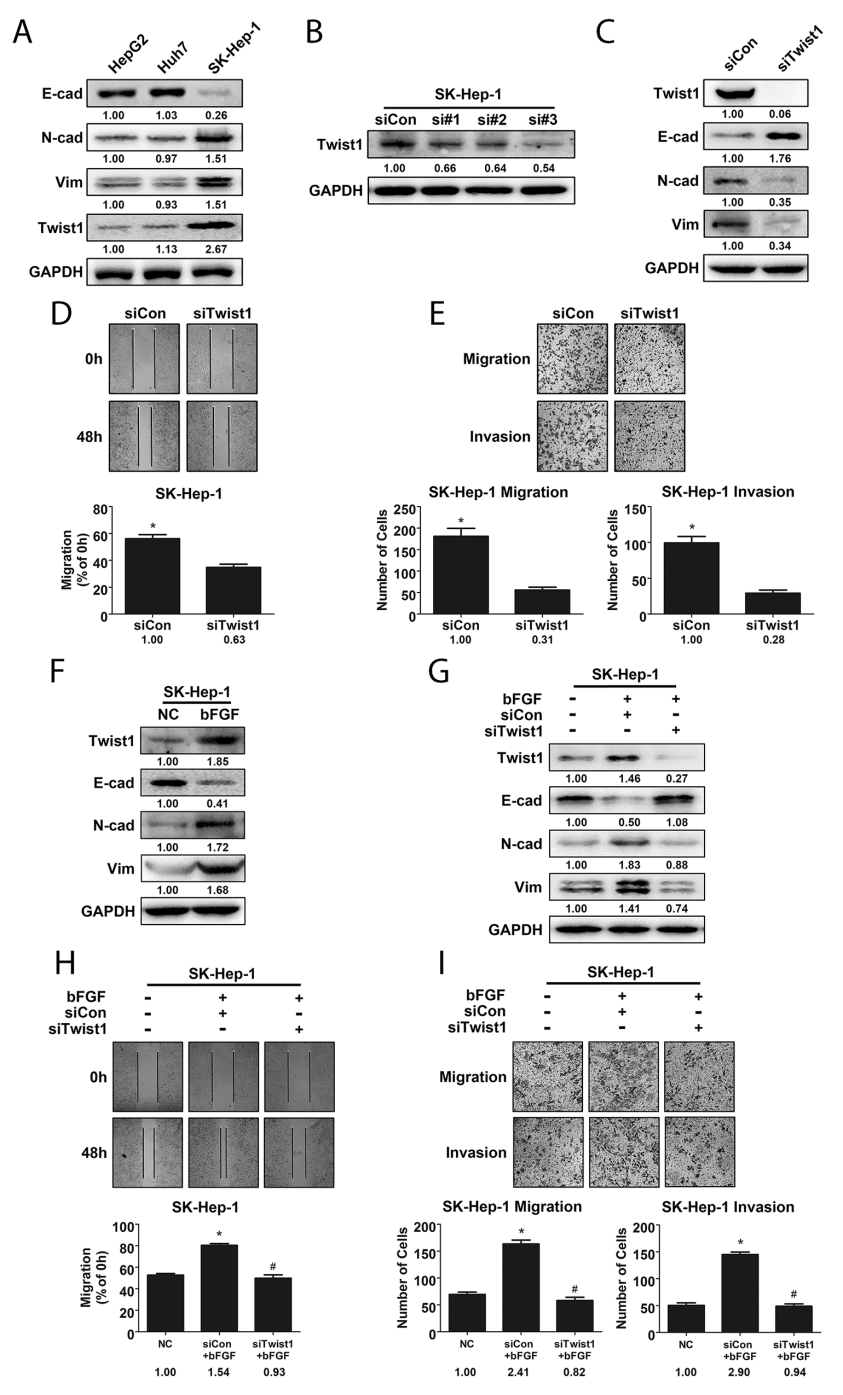
Twist1 played a crucial role in bFGF-induced EMT of HCC cells **(A)** Highly metastatic cell line SK-Hep-1 exhibited a higher extent of EMT and expressed more Twist1. **(B)** Knockdown efficiency of Twist1 siRNA in SK-Hep-1 cells was validated by western blotting. **(C)** E-cad, N-cad and Vimentin levels were detected by western blotting after silencing Twist1 in SK-Hep-1 cells. The metastatic potential of SK-Hep-1 cells after Twist1 knockdown was evaluated by wound healing **(D)** and migration and invasion assay **(E)**. **(F)** bFGF upregulated Twist1 expression in SK-Hep-1 cells. **(G)** Western blotting was used to validate cell phenotypes associated with EMT in Twist1-knockdown SK-Hep-1 cells treated with bFGF. The metastatic potential of Twist1-knockdown SK-Hep-1 cells treated with bFGF was evaluated by wound healing **(H)** and migration and invasion assay **(I)**, and Twist1 was found to be important for EMT induced by bFGF. ^*^p<0.05 compared with control, ^#^p<0.05 compared with bFGF.

Twist1, a basic helix-loop-helix transcription factor, has been demonstrated as an important EMT marker in many cancers [19, 20]. In this study, we compared the differences in expression of cell phenotypes and of Twist1 in HCC cell lines with varying metastatic potential. As expected, the highly invasive SK-Hep-1 cells had higher levels of expression of Vimentin and N-cadherin compared to HepG2 and Huh7 cells, while E-cadherin expression was lower in SK-Hep-1 cells (Figure 3A). Further, we confirmed the role of Twist1 in EMT with small interfering RNA. After introducing 3 Twist1-specific siRNAs and control-siRNA respectively into SK-Hep-1 cells for 24 h, we measured Twist1 expression and selected siRNA#2 and siRNA#3 to knock down Twist1 (Figure 3B), and we found that E-cadherin was upregulated and Vimentin and N-cadherin were downregulated with knockdown of Twist1 (Figure 3C, Supplementary Figure 1A). In addition, knockdown of Twist1 significantly decreased the extent of wound closure and number of migrated/invaded SK-Hep-1 cells (Figure 3D-3E and Supplementary Figure 1B-1C). These data verified that Twist1 promoted EMT in hepatocellular carcinoma cells.

To prove whether Twist1 is a key modulator of EMT induced by bFGF, first, we detected Twist1 expression after bFGF treatment, indeed, bFGF enhanced Twist1 expression accompanied with E-cadherin downregulation and Vimentin and N-cadherin upregulation (Figure 3F). Then, SK-Hep-1 cells with Twist1 knockdown were treated with bFGF. We found that silencing Twist1 reversed the induction of EMT by bFGF in SK-Hep-1 cells. With Twist1 knockdown and bFGF treatment, the expression of N-cadherin and Vimentin, number of migrated and invaded cells and extent of wound closure were decreased, while E-cadherin expression was increased (Figure 3G-3I, Supplementary Figure 1D-1F). These results showed that Twist1 was crucial for bFGF-induced EMT in liver cancer.

### bFGF induced EMT by enhancing the stability of Twist1

**Figure 4:**
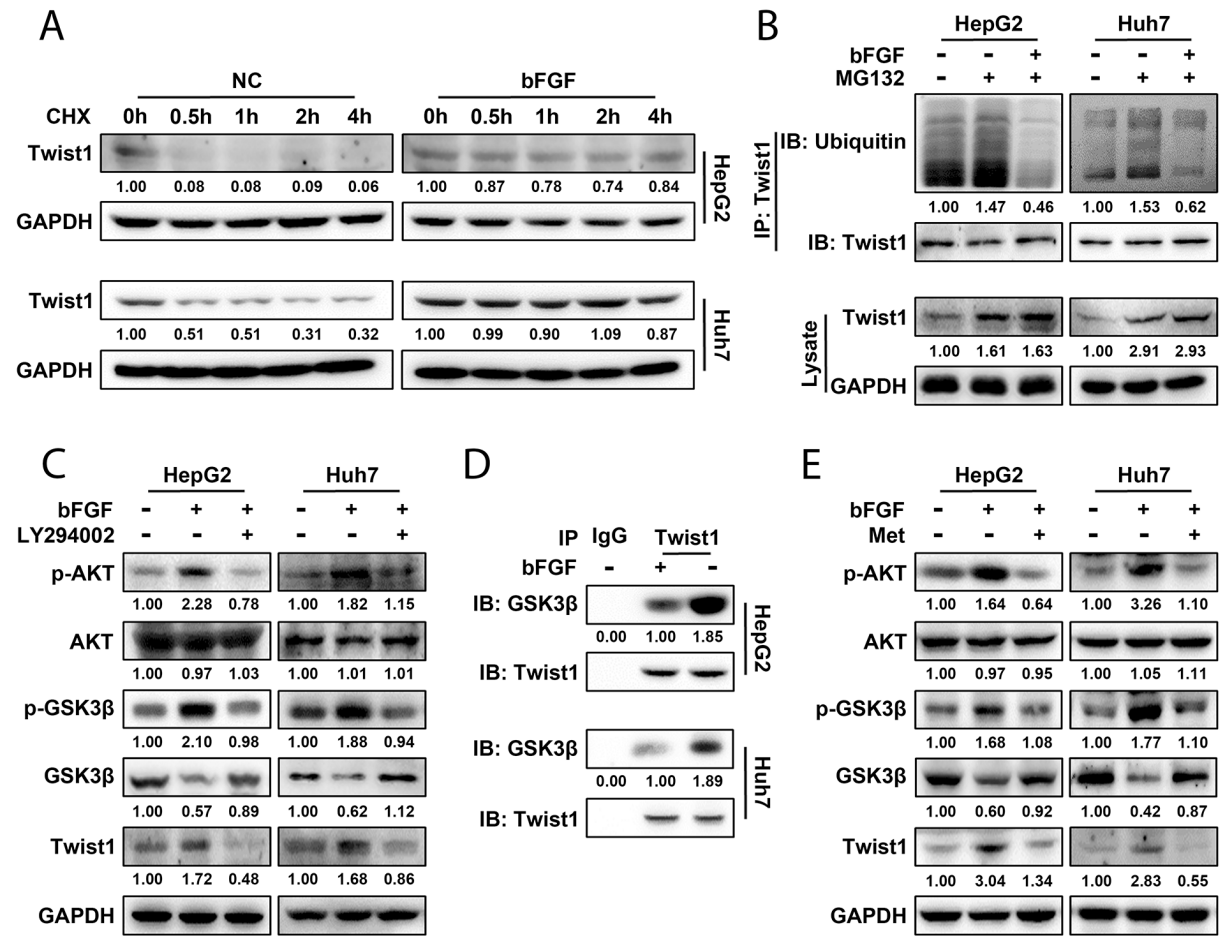
bFGF induced EMT in HepG2 and Huh7 cells by enhancing the stability of Twist1 through the AKT/GSK-3β signalling pathway **(A)** Twist1 expression was detected by western blotting after bFGF treatment or not. **(B)** After HepG2 and Huh7 cells were treated with MG132 or a combination of MG132 and bFGF, ubiquitin was immunoprecipitated by Twist1, and its expression was detected by western blotting. **(C)** After treatment with bFGF or bFGF and LY294002 (20 ng/ml), p-AKT, AKT, p-GSK3β, GSK-3β and Twist1 expression was evaluated by western blotting. **(D)** After treating HepG2 and Huh7 cells with bFGF for 48 h, GSK-3β was immunoprecipitated by Twist1, and its expression was detected by western blotting. **(E)** After treatment with bFGF or bFGF and metformin together, p-AKT, AKT, p-GSK3β, GSK-3β and Twist1 expression was assessed by western blotting.

As a transcription factor, Twist1 is generally unstable in the cytoplasm and is quickly degraded through the ubiquitin-proteasome pathway [30]. Thus, we detected the changes in Twist1 stability in response to bFGF treatment. After treatment of cells with cycloheximide to repress protein synthesis, Twist1 expression was found to be higher in cells treated with bFGF than in cells without treatment at every time point, indicating that bFGF inhibited Twist1 degradation in a time-dependent manner (Figure 4A).

Furthermore, we investigated whether the change in Twist1 in response to bFGF stimulation was regulated by the ubiquitination-proteasome degradation pathway. Using MG132 to inhibit proteasomal activity, ubiquitination of Twist1 increased compared to the control, whereas Twist1 ubiquitination was downregulated significantly with combined treatment of bFGF and MG132 compared treatment with MG132 alone. In addition, Twist1 expression was upregulated in cells treated with MG132 irrespective of bFGF in comparison with that in untreated cells (Figure 4B). This indicated that bFGF participated in regulating ubiquitin-mediated degradation of Twist1. These data demonstrated that bFGF could inhibit ubiquitination of Twist1, thereby enhancing the stabilization of Twist1 in HepG2 and Huh7 cells.

### bFGF enhanced Twist1 stability through the activation of AKT/GSK-3β signalling

In AKT/GSK-3β signalling pathway, AKT could inhibit GSK-3β activity and promote Snail protein stability [25] and participate in metastasis of prostate cancer. Hence, we next studied whether the AKT/GSK-3β pathway participated in the promotion of EMT by bFGF in HepG2 and Huh7 cells. We observed that phosphorylated AKT (p-AKT) and phosphorylated GSK-3β (p-GSK3β) were significantly upregulated, whereas the expression of total AKT was not changed, and GSK-3β was downregulated after induction of EMT by bFGF. After treating HepG2 and Huh7 cells with a combination of bFGF and the PI3K inhibitor LY294002 that could inhibit AKT phosphorylation (20 ng/ml) for 48 h, we measured the expression and phosphorylation of AKT and GSK-3β. As we expected, the enhanced phosphorylation of AKT and GSK-3β by bFGF was significantly reversed by the LY294002 (Figure 4C). This indicated that bFGF could activate the AKT/GSK-3β pathway. We further assessed the relationship between the AKT/GSK-3β pathway and Twist1 expression. In response to bFGF treatment, Twist1 expression was upregulated along with an increase in the phosphorylation level of AKT and GSK-3β, but the phenomenon disappeared when the cells were stimulated with a combination of LY294002 and bFGF (Figure 4C). This illustrated that bFGF upregulated Twist1 by activating AKT/GSK-3β signalling.

Twist1 is easily degraded in the cytoplasm, while the active form, non-phosphorylated GSK-3β, can combine with c-Jun and promote the degradation of c-Jun [31]. We hypothesized that the AKT/GSK-3β pathway influenced Twist1 expression through a direct or an indirect association between GSK-3β and Twist1. We used anti-Twist1 antibody to immunoprecipitate GSK-3β and demonstrated that direct or indirect association between GSK-3β and Twist1 was markedly decreased in cells treated with bFGF compared to control cells (Figure 4D). This indicated that GSK-3β had a specific impact on Twist1, and the interaction between them promoted Twist1 ubiquitination. Collectively, these data suggest that bFGF could activate the AKT/GSK-3β pathway and reduce the interaction between GSK-3β and Twist1 leading to ubiquitination and degradation decrease of Twist1.

### Metformin reverses bFGF induced EMT by inhibition of the AKT/GSK-3β pathway

To further explore the mechanism of the reversion of bFGF-induced EMT by metformin in HepG2 and Huh7 cells, we measured the status of the AKT/GSK-3β pathway after combined treatment with bFGF and metformin to confirm whether metformin exerts its inhibitory effect on bFGF through AKT/GSK-3β signalling. Similar to the combined treatment of bFGF and LY294002, after the treatment of bFGF and metformin for 48 h, the increase in p-AKT and p-GSK-3β by bFGF disappeared, whereas the expression of total AKT was not changed, and GSK-3β was upregulated, suggesting that metformin suppressed the activation of the AKT/GSK-3β pathway mediated by bFGF. Meanwhile, as shown in Figure 4E, Twist1 expression decreased along with the suppression of the AKT/GSK-3β pathway by metformin. In addition, we further found that metformin reversed the inhibition of GSK-3β by bFGF. This was consistent with the proteomics data, which showed that metformin upregulated GSK-3β (Supplementary Table 1). This indicated that metformin downregulated Twist1 by repressing the AKT/GSK-3β pathway. Collectively, these results indicated that metformin could abolish the induction of EMT in HepG2 and Huh7 cells by bFGF through the same signalling pathway.

## DISCUSSION

HCC is the second leading cause of cancer mortality the fifth most common type of solid tumour worldwide [1], its treatment is still challenging, which sparked considerable interest in this disease. In this study, using quantitative proteomics, we analysed differences in protein expression between metformin treatment and control treatment in HCC cells. We totally quantified 2707 proteins and among these, 52 proteins upregulated while 378 proteins downregulated. By the analysis of proteomics data sets with the IPA tool, we further assessed changes in signalling pathways of cells under metformin treatment. Interestingly, we found that FGF signalling, which has a potential association with metastasis, was inhibited after metformin stimulation. In HCC, the high rate of metastasis is still one of the most important reasons for high recurrence and mortality and has been the bottleneck in therapies. The presence of metastasis takes away the opportunity for patients to undergo surgery, which is the most effective therapy for HCC.

bFGF, the FGFR activator, was shown to affect tumour progression *in vitro* and *in vivo* [32]. Clinically, serum bFGF levels of HCC patients were significantly higher than those of healthy volunteers [33], while *in vitro*, bFGF could promote EMT in LH86 cells, a human HCC cell line [26]. EMT is one of the most important mechanisms in metastasis by which epithelial cancer cells transdifferentiate to mesenchymal cells and become motile [11]. Therefore, we focused on whether bFGF could induce EMT in HCC cells. In this study, we demonstrated that bFGF induced HCC cell lines HepG2 and Huh7 to change from epithelial to mesenchymal phenotype and enhanced their migratory and invasive abilities.

Several transcription factors have been reported to regulate EMT. Twist1 is a basic helix-loop-helix transcription factor that is known as a crucial marker of EMT. It can downregulate E-cadherin by binding with the E-box in the E-cadherin gene promoter resulting in the initiation of EMT [20]. We found that Twist1 expression was markedly higher in SK-Hep-1 cells than that in HepG2 and Huh7 cells, while compared to SK-Hep-1, HepG2 and Huh7 cells possessed lower metastatic potential. Furthermore, SK-Hep-1 cells expressed higher levels of mesenchymal markers. Meanwhile, EMT was suppressed after Twist1 knockdown by siRNA in SK-Hep-1 cells. Furthermore, Twist1 knockdown reversed EMT induced by bFGF, and the increase in migratory and invasive abilities of SK-Hep-1 cells treated with bFGF was abolished after the siRNA-mediated interference of Twist1. These results suggested that Twist1 is a crucial factor in EMT induced by bFGF in liver cancer cells.

Next, we attempted to determine the mechanism of EMT induction by bFGF mediated via Twist1 expression. As a transcription factor, Twist1 primarily exists and functions in the nucleus. In the cytoplasm, Twist1 has much lower activity than that in the nucleus and is quickly degraded through the ubiquitin-proteasome system [30]. Hence, we hypothesized that bFGF impacted the stability of Twist1 through the ubiquitin-proteasome system. In response to treatment with cycloheximide, the Twist1 degradation rate was slower in the bFGF treatment group than that in the control group. As Twist1 degradation depends on the ubiquitin-proteasome system [30], we further compared the Twist1 ubiquitination level between the bFGF treatment and control groups. By inhibiting proteasome with MG132, we found that the ubiquitination of Twist1 was downregulated by bFGF, suggesting that bFGF prevented the degradation of Twist1 in accordance with our prediction.

**Figure 5:**
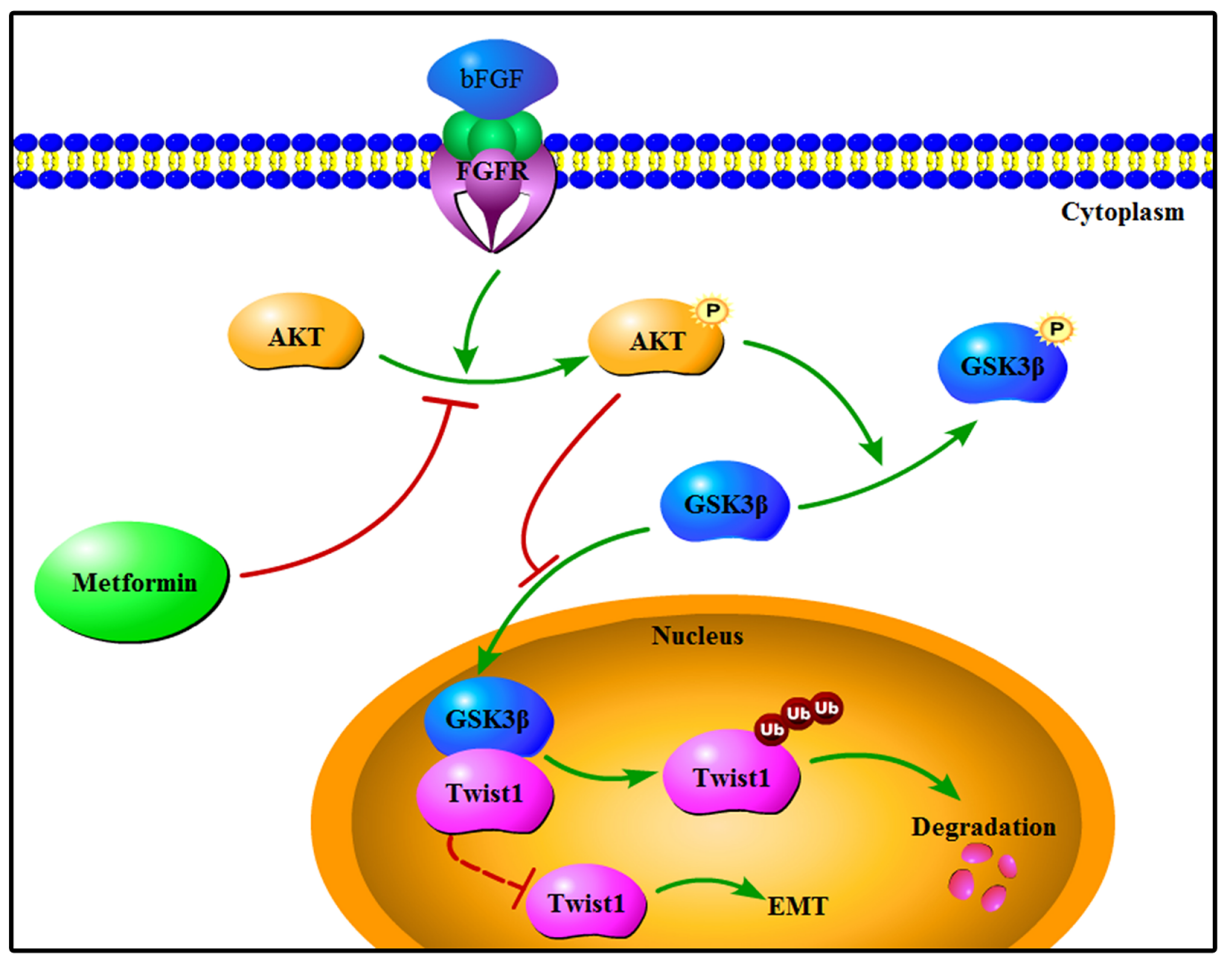
Schematic representation of reversion of bFGF-induced EMT by metformin in HepG2 and Huh7 cells

These results demonstrated that bFGF decreased Twist1 ubiquitination leading to its enhanced stabilization and accumulation. Then, we wondered which signalling pathway was activated or suppressed by bFGF that resulted in the increased accumulation of Twist1 and initiation of EMT. bFGF has been shown to promote phosphorylation of AKT and GSK-3β in the prostate cancer cell line PC-3 [25]. In this study, consistent with the results of Liu et al., we found that the phosphorylation of AKT and GSK-3β was increased by stimulation of bFGF in HepG2 and Huh7 cells, and the expression of GSK-3β was decreased. This suggested that bFGF may promote EMT through the AKT/GSK-3β signalling pathway. We selected LY294002 to inhibit AKT phosphorylation to determine the role of bFGF in AKT/GSK-3β signalling. LY294002 significantly reversed the bFGF-mediated activation of the AKT/GSK-3β pathway accompanying the reduction in expression of Twist1. A previous study showed that GSK-3β could bind to Snail and inhibit its degradation [34]. Hence, we further confirmed that bFGF activated the AKT/GSK-3β pathway, leading to inactivation of GSK-3β and the subsequent decrease in interaction between GSK-3β and Twist1 due to the increase of the inactive phosphorylated GSK-3β. We speculate that there must be some interactions between GSK-3β and Twist1, which enables GSK-3β to facilitate Twist1 ubiquitination and weaken its stability, while bFGF likely decreases the association between GSK-3β and Twist1 through phosphorylating GSK-3β to deactivate GSK-3β by activating the AKT/GSK-3β pathway and eventually induces EMT in HepG2 and Huh7 cells (Figure 5).

Accumulating epidemiological evidence reveals that the incidence and malignancy of HCC is markedly higher in patients with T2DM [34], while metformin, a biguanide derivative that is widely used to treat T2DM in the clinic, has been reported play an important role in sensitizing cancer cells to chemotherapy [35] and inducing apoptosis in cancer cells [36]. Based on our previous study on the use of metformin in HCC and the proteomics results in this study, we determined the induction of EMT in liver cancer cells by bFGF, and we confirmed that metformin could reverse the bFGF-induced EMT in HepG2 and Huh7 cells. In addition, we demonstrated that metformin repressed the AKT/GSK-3β pathway, the same signalling pathway activated by bFGF during EMT, to inhibit the migration and invasion of HCC cells, which supported the use of metformin in the clinical therapy of HCC.

In conclusion, our study demonstrated that bFGF could promote EMT in HCC and clarified the new pathway involving AKT/GSK-3β/Twist1 signalling in EMT induced by bFGF. Moreover, we illustrated that metformin reversed the effect of bFGF on the EMT of HCC through the same signalling pathway. These findings suggest that the antidiabetic drug metformin has a new role that could be used in HCC treatment to control metastasis and improve prognosis.

## MATERIALS AND METHODS

### Chemicals and reagents

Metformin, LY294002 and cycloheximide were obtained from Sigma-Aldrich (St. Louis, MO, USA). MG132 was purchased from Selleckchem (Houston, TX, USA). Recombinant human basic fibroblast growth factor (bFGF) was purchased from PeproTech (London, UK). The following antibodies were used in this study: anti-E-cadherin, anti-N-cadherin, anti-AKT, anti-phosphorylated-AKT were obtained from Abcam (Cambridge, MA, USA); anti-Vimentin, anti-GSK-3β, anti-phosphorylated-GSK-3β, anti-Twist1 and anti-GAPDH were obtained from Proteintech (Rosemont, IL, USA). Peroxidase-conjugated polyclonal goat anti-rabbit and polyclonal goat anti-mouse IgG antibodies were purchased from Thermo-Pierce (Rockford, IL, USA).

### Peptide dimethyl labelling and LC-MS

Proteins were extracted from HCC cells treated with or without metformin, and the extracted protein mixtures were digested into peptides by trypsin and labelled using the in-solution stable isotope dimethyl labelling method [37]. The metformin treatment group was labelled with deuterium and the no treatment group was labelled with hydrogen. The dimethyl-labelled peptides were fractionated using an HPLC system fitted with an SCX column. Peptide fractions were analysed with an LTQ-Orbitrap Velos mass spectrometer equipped with a nanoLC interface. Data sets representing altered expression of proteins derived from quantitative proteomics were categorized into and analysed with the Ingenuity Pathway Analysis Tool (Ingenuity Systems, USA; http://www.ingenuity.com/).

### Cell culture

The human hepatocellular carcinoma cell lines HepG2, Huh7 and SK-Hep-1 were obtained from the tumour cell bank of the Chinese Academy of Medical Science. Cells were cultured in RPMI-1640 medium (Gibco-BRL, Carlsbad, CA, USA) supplemented with 10% foetal bovine serum (Gibco-BRL, Carlsbad, CA, USA), 100 μg/ml penicillin and 100 μg/ml streptomycin (Invitrogen; Life Technologies, Carlsbad, CA, USA) in an incubator with 5% CO_2_ at 37°C.

### RNA interference

Cells (2 x 10^5^) were seeded in 6-well plates, cultured for 24 hours and then transfected with 100 pmol siRNA using Lipofectamine 2000 (Invitrogen; Life Technologies, Carlsbad, CA, USA) in serum-free medium according to the manufacturer′s protocol. The medium was changed to complete culture medium after 4 hours. After another 24 hours of incubation in a 37°C incubator, the cells were harvested or used for other experiments. All siRNAs were obtained from GenePharma (Shanghai, China), the three Twist1 siRNA sequences were: human Twist1 siRNA#1, sense 5′- CCUGAGCAACAGCGAGGAATT-3′ and antisense 5′-UUCCUCGCUGUUGCUCAGGTT-3′; siRNA#2, sense 5′- GCAAGAUUCAGACCCUCAATT-3′ and antisense 5′- UUGAGGGUCUGAAUCUUGCTT-3′; siRNA#3, sense 5′- GAUGGCAAGCUGCAGCUAUTT-3′ and antisense 5′- AUAGCUGCAGCUUGCCAUCTT-3′.

### Wound healing or scratch assay

Cells were plated in triplicate in 6-well cell culture plates until they grew to confluency. A 200-μl pipette tip was used to make the artificial wound across the wells. The cell debris was washed 3 times with phosphate-buffered saline (PBS). The normal control cells were not subjected to any treatment, and the other two groups of cells were treated with bFGF or bFGF and metformin without FBS, we selected 20 ng/ml bFGF to induce EMT [25] and 10 μmol/ml metformin we used in previous work [36]. The cells were maintained in an incubator for 48 hours. Cell migration was observed with a microscope and photographed, and the wound width was measured for comparison.

### Cell migration and invasion assays

Cell migration and invasion assays were performed using transwell chambers fitted with polycarbonate filters (8-μm pore size, Corning). For the invasion assay, the filter was pre-coated with Matrigel (BD Biosciences, Franklin Lakes, NJ, USA). Cells were pre-treated with or without bFGF and metformin, and after 48 hours, the cells (2×10^5^) were harvested and seeded in transwell chambers filled with 300 μl culture medium containing 0.1% FBS, and the cells were treated as described above. The lower chambers were filled with 600 μl medium containing 10% FBS. After 48 hours of incubation, for the migration assay, cells had migrated through the filters and adhered on the lower surface of the filter membranes. The adherent cells were fixed with 4% paraformaldehyde and stained with 0.1% crystal violet. For the invasion assay, the Matrigel matrix on the upper surface of the filters was removed before staining. For both migration and invasion assays, the final step involved counting the cells under a microscope.

### Cell lysate preparation and protein extraction

Cells were all harvested with cell scrapers after washing 3 times with pre-cold PBS. Then, the harvested cells were centrifuged for 3 minutes at 1000x g, and the pellets were saved. The cell pellets were lysed with RIPA lysis buffer (KeyGen Biotech, Shanghai, China) containing 1 mmol/l phenylmethylsulphonyl fluoride (KeyGen Biotech, Shanghai, China) and 1 mmol/l phosphatase inhibitor cocktail (KeyGen Biotech, Shanghai, China). The mixture was centrifuged for 10 minutes at 12000x g, and the supernatant containing proteins was saved.

### Western blotting analysis

Total protein was diluted to a final concentration of 4 mg/ml with ultrapure water and loading buffer (KeyGen Biotech, Shanghai, China). Equal amounts of protein samples were subjected to 8 or 10% sodium dodecyl sulphate polyacrylamide gel electrophoresis (SDS-PAGE) to separate the proteins based on their molecular weights. The proteins were transferred to 0.4-mm PDVF membranes (Bio-Rad, USA). The membranes were then blocked with 5% skim milk mixed with Tris-buffered saline with 0.1% Tween 20 (pH 6.8; TBST) for 2 hours at room temperature. The membranes were incubated with primary antibodies (1:1000) and secondary antibodies (1:5000). Finally, the bands were detected by chemiluminescence.

### Co-immunoprecipitation (co-IP) assay

Co-immunoprecipitated assay used a Pierce Co-Immunoprecipitation (Co-IP) Kit, purchased from Thermo Scientific Inc. (Rockford, IL, USA), following the manufacturer’s protocol. Briefly, protein samples were prepared as described above. Anti-Twist1 primary antibody (10 μg) was immobilized onto AminoLinkPlus Coupling Resin for 2 hours at room temperature, and then the protein samples were added to the resin containing the primary antibody and incubated overnight at 4°C. The resin was washed with elution buffer to elute the protein bound to the anti-Twist1 primary antibody. The protein eluted from the resin was analysed by western blotting.

### Statistical analysis

SPSS 17.0 software was used for statistical analysis (IBM, USA). All experiments were repeated 3 times. The values were presented as the means ± standard deviation. Statistical analyses were performed using Student’s *t*-test, and one-way ANOVA was used to analyse variance. A p-value < 0.05 represented statistical significance.

## SUPPLEMENTARY MATERIALS FIGURE AND TABLE





## Abbreviations

(HCC): Hepatocellular carcinoma
(bFGF): Basic Fibroblast Growth Factor
(EMT): Epithelial-mesenchymal Transition
(AKT): Protein Kinase B
(GSK-3β): Glycogen Synthase Kinase-3β
(Co-IP): Co-Immunoprecipitation
