# Supplementary material for: Metformin reverses bFGF-induced epithelial-mesenchymal transition in HCC cells
Source: Oncotarget. 2017 Oct 31;8(61):104247–57. doi: 10.18632/oncotarget.22200 (PMC5732803; doi:10.18632/oncotarget.22200)
Supplement: Supplementary file 1 [file oncotarget-08-104247-s001.pdf]

Metformin reverses bFGF-induced epithelial-mesenchymal transition in HCC cells

SUPPLEMENTARY MATERIALS

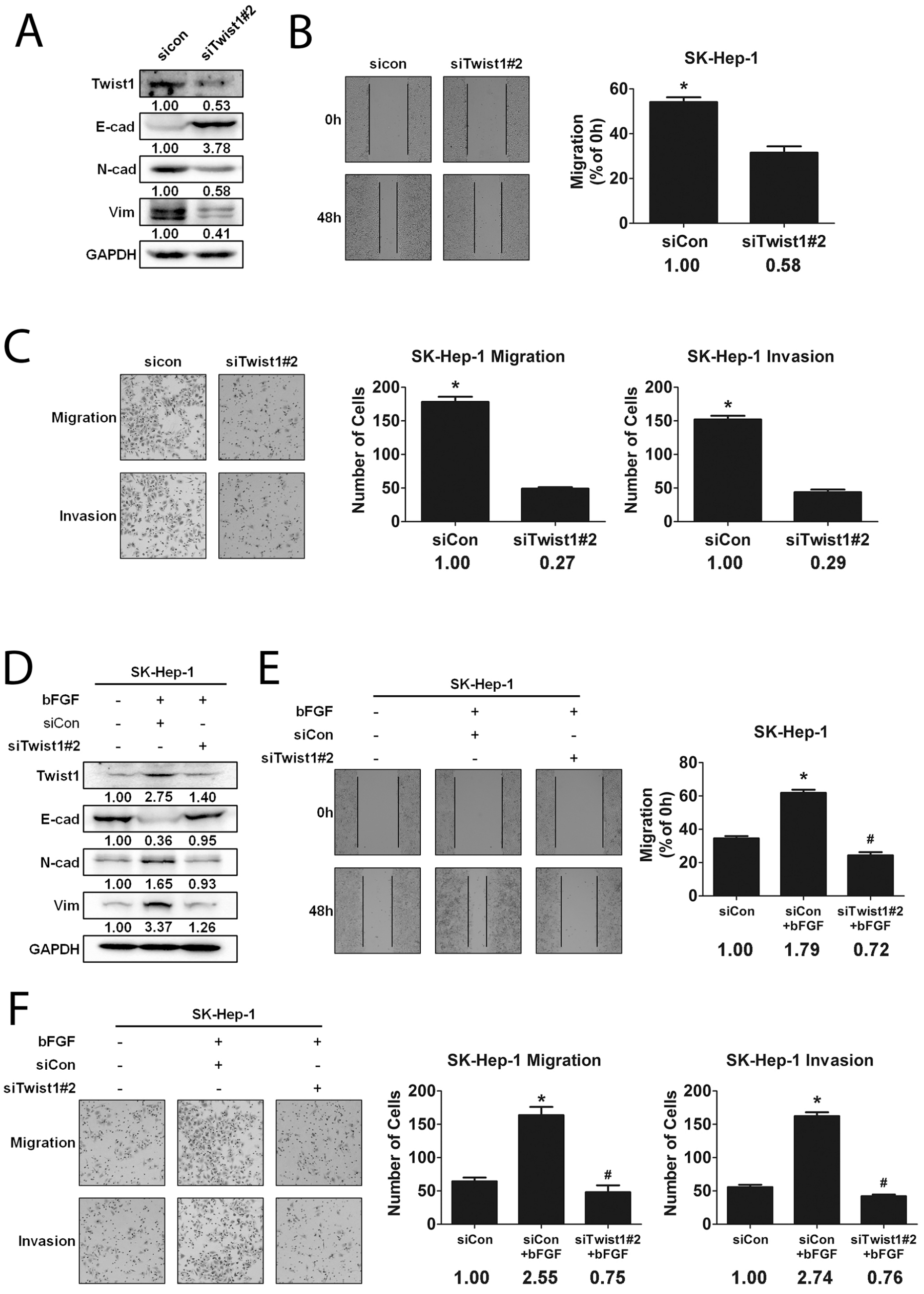

Supplementary Figure 1: Twist1 played a crucial role in bFGF-induced EMT of HCC cells. (A) E-cad, N-cad and Vimentin levels were detected by western blotting after silencing Twist1 in SK-Hep-1 cells. The metastatic potential of SK-Hep-1 cells after Twist1 knockdown was evaluated by wound healing **(B)** and migration and invasion assay **(C)**. **(D)** Western blotting was used to validate cell phenotypes associated with EMT in Twist1-knockdown SK-Hep-1 cells treated with bFGF. The metastatic potential of Twist1-knockdown SK-Hep-1 cells treated with bFGF was evaluated by wound healing **(E)** and migration and invasion assay **(F)**, and Twist1 was found to be important for EMT induced by bFGF. $^{*}p<0.05$ compared with control, $^{\#}p<0.05$ compared with bFGF.

Supplementary Table 1: Quantitative proteomics data of HCC cells with or without metformin treatment.

See Supplementary File 1
